# Implant Soft-Tissue Attachment Using 3D Oral Mucosal Models—A Pilot Study

**DOI:** 10.3390/dj8030072

**Published:** 2020-07-07

**Authors:** Emilia Barker, Lina AlQobaly, Zahab Shaikh, Kirsty Franklin, Keyvan Moharamzadeh

**Affiliations:** School of Clinical Dentistry, University of Sheffield, Western Bank, Sheffield S10 2TN, UK; emilia.barker@sheffield.ac.uk (E.B.); lalqobaly1@sheffield.ac.uk (L.A.); zahabnoshad@yahoo.com (Z.S.); K.L.Franklin@sheffield.ac.uk (K.F.)

**Keywords:** oral mucosa, tissue engineering, implant, soft tissue attachment, titanium, zirconia

## Abstract

Purpose: The aim of this study was to investigate soft-tissue attachment to different metal, ceramic, and polymer implant surfaces using an inflamed, three-dimensional (3D), tissue-engineered, human oral mucosal model, as well as multiple-endpoint qualitative and quantitative biological approaches. Methods: Normal human oral fibroblasts, OKF6/TERT-2 keratinocytes and THP-1 monocytes were cultured, and full-thickness, 3D oral mucosal models were engineered inside tissue culture inserts. Sand-blasted and acid-etched (SLA) and machined (M) titanium–zirconium alloy (TiZr; commercially known as Roxolid; Institut Straumann AG, Switzerland), ceramic (ZrO_2_), and polyether ether ketone (PEEK) rods (Ø 4 mm × 8 mm) were inserted into the center of tissue-engineered oral mucosa following a Ø 4mm punch biopsy. Inflammation was simulated with addition of the lipopolysaccharide (LPS) of *Escherichia coli* (E. coli) and tumor necrosis factor (TNF)-alpha to the culture medium. Implant soft-tissue attachment was assessed using histology, an implant pull-test with PrestoBlue assay, and scanning electron microscopy (SEM). Results: Inflamed, full-thickness, 3D human oral mucosal models with inserted implants were successfully engineered and histologically characterized. The implant pull-test with PrestoBlue assay showed higher viability of the tissue that remained attached to the TiZr-SLA surface compared to the other test groups. This difference was statistically significant (*p* < 0.05). SEM analysis showed evidence of epithelial cell attachment on different implant surfaces. Conclusions: The inflamed, 3D, oral mucosal model has the potential to be used as a suitable in vitro test system for visualization and quantification of implant soft-tissue attachment. The results of our study indicate greater soft tissue attachment to TiZr-SLA compared to TiZr-M, ceramic, and PEEK surfaces.

## 1. Introduction

The long-term success of dental implants not only requires a sustained osseointegration, but also the integrity of a biological seal formed by the peri-mucosal tissue surrounding the implants. Investigations have been carried out to establish stable implant–soft tissue attachment. One of the promising strategies is through the modification of the implant surfaces. It has been proven by studies that the macroscopic surface topography and microscopic surface roughness influence the interaction between implants and surrounding soft tissues [1].

Different study models have been used to examine and evaluate the implant–soft tissue interface [2]. In general, animal models and some in vitro studies had been used for this purpose. Commonly used in vitro techniques for the evaluation of the implant–soft tissue interaction are primarily based on monolayer culture of keratinocytes on titanium surfaces [3,4], which do not mimic the in vivo situation. To be able to obtain an accurate evaluation, the in vitro test model must resemble the clinical situation as closely as possible.

Over the past decade, we have developed and characterized several tissue-engineered models of human oral mucosa for various in vitro applications [5,6]. Initially, a three-dimensional (3D) human oral mucosal model was developed for biocompatibility assessment of restorative dental materials, including dental composite resins [7,8], and for the biological evaluation of oral care products, including antiseptic mouthwashes [9].

The 3D model gives a more realistic picture of the biological effects of the tested materials on human oral mucosa, and allows us to study multiple responses of the oral mucosa to different stimuli.

We were the first research group to develop a novel, 3D transmucosal model based on tissue-engineered human oral mucosa for implant–soft tissue interface assessment [10]. The 3D model was designed to assess the interaction between dental implants and human oral mucosa in terms of the implant–soft tissue attachment, biological seal, and the interface contour [10]. Our experiments on the 3D model have shown that the results obtained from multiple-endpoint analysis of the oral mucosa model were more clinically relevant and more informative than monolayer cell culture systems [11,12,13].

The 3D in vitro model enabled examination of the implant–soft tissue interface using several qualitative and quantitative biological endpoints, including basic histology using light microscopy (LM), ultrastructural analysis by scanning electron microscopy (SEM) and transmission electron microscopy (TEM) [14], confocal laser scanning microscopy (CLSM), a soft tissue–implant adhesion test [10], contour analysis of the implant–soft tissue interface [13], and measurement of the biological seal of implant–soft tissue interface [12].

Further studies have included optimization of 3D oral mucosal models to investigate bacterial invasion of human gingival mucosa, with a specific focus on periodontal pathogens like *Porphyromonas gingivalis* [15]. This model has enabled quantification of the release of various pro-inflammatory cytokines and chemokines by the 3D oral mucosal model in response to exposure to *P. gingivalis*.

The 3D oral mucosa model was further validated by comparing its response to various concentrations of ethanol to those of fresh clinical biopsies of human oral mucosa and monolayer cultures of epithelial cells [16]. In our previous study, oral mucosa models, monolayer cell cultures, and fresh oral mucosa biopsies were exposed to increasing concentrations of ethanol from 5% to 50% for 30 and 60 s. Basic histological assessment and the Alamar Blue tissue viability assay were carried out to record the biological response. The results showed that the effects of ethanol on the 3D oral mucosa models were similar to those on the fresh human tissue biopsies, and the 3D oral mucosa model detected the concentration-dependent and time-dependent biological effects of ethanol. This study indicated that the 3D oral mucosal model could be used as a more clinically relevant model to test different biomaterials, such as dental implants, compared to the monolayer cell culture systems.

Advanced tissue-engineered models of human alveolar bone and oral mucosa have been developed using conventional [17,18] and 3D printing technologies [19]. Tissue-engineered oral mucosa has also been used recently to assess gingival soft-tissue attachment to a biomodified glass ionomer cement (GIC) [20], and for evaluation of the biological effects of electronic cigarettes [21].

The aim of this study was to investigate soft-tissue attachment to different metal, ceramic, and polymer implant surfaces using an inflamed, 3D, tissue-engineered human oral mucosal model; multiple-endpoint qualitative methods, including histology and scanning electron microscopy; and a quantitative biological approach with a tissue viability pull test. This model differs from the previous models, because in addition to oral fibroblasts and keratinocytes, it contains immune cells capable of producing an inflammatory response induced by bacterial endotoxins. Simulation of inflammation is an important and novel aspect of this study, as implant placement induces significant damage to the oral mucosa and creates a potential entry route for oral microorganisms. This can provoke an inflammatory response, which would be present following the initial implant placement and during early healing, and may affect soft-tissue attachment to the implant and abutment surface.

## 2. Materials and Methods

### 2.1. Cell Source

Normal human oral fibroblasts were obtained from the frozen stocks in the laboratories of the School of Clinical Dentistry at University of Sheffield. The cells had been obtained in the past from healthy patients having minor oral surgery, with their written informed consent, at Charles Clifford Dental Hospital, under an appropriate research ethics permission from the U.K. National Research Ethics Services Committee (Reference number 15/LO/0116) (date 21/01/2015).

OKF6/TERT-2 cells, which are immortalized human oral keratinocyte cell lines, were kindly provided by Brigham and Women’s Hospital, Harvard Institute of Medicine, United States.

The THP-1 monocyte cell line was purchased from Sigma Aldrich, Dorset, United Kingdom. Although these cancerous cells are derived from acute monocytic leukemia, they are frequently used for in vitro biocompatibility assessment of dental materials, as the cell lines have the advantage of being reproducible, and prevent batch-to-batch variations compared to primary cells [5].

### 2.2. Cell Culture

The oral fibroblasts were cultured in high-glucose Dulbecco’s modified Eagle’s medium (DMEM) (Sigma, Dorset, United Kingdom), supplemented with 2% L-glutamine (Sigma, Dorset, United Kingdom), 100 IU/100 mg ml^-1^ penicillin/streptomycin (Sigma, Dorset, United Kingdom), and 10% fetal calf serum (FCS) (Sigma, Dorset, United Kingdom).

The OKF6/TERT-2 human oral keratinocyte cells were cultured in Green’s medium, which consists of Dulbecco’s modified Eagle’s medium (DMEM) and Ham’s F12 medium in a 3:1 ratio, and is supplemented with 10% fetal calf serum (FCS), 10 ng/mL epidermal growth factor, 0.4 µg/mL hydrocortisone, 0.1 mM adenine, 5 µg/mL insulin, 5 µg/mL transferrin, 0.2 µM triiodothyronine, 2 mg/mL L-glutamine, 50 U/mL penicillin, and 50 U/mL streptomycin. All the agents were purchased from Sigma-Aldrich (Dorset, United Kingdom). Since live bacteria were not used in this study, the use of antibiotics in the cell culture medium would have minimal impact on the results.

The THP-1 monocytes were cultured in RPMI 1640 culture medium (Sigma, Dorset, United Kingdom) supplemented with 2 mM L-glutamine and 10% FCS.

Cultures were maintained in the incubators at 37 °C and 5% CO_2_. The cells were cultured until 80–100% confluency was achieved.

### 2.3. Materials

Four types of rods with 4 mm diameter and 8 mm length were fabricated and tested in this study:

- TiZr-SLA: titanium–zirconium alloy, which underwent sandblasting and acid etching (SLA), according to the manufacturer’s protocol, and was gamma sterilized;
- TiZr-M: as-machined TiZr (gamma sterilized);
- ZrO_2_-M: as-machined ZrO_2_ (zirconia), which was ethylene oxide-sterilized;
- PEEK-M: as-machined PEEK (polyether ether ketone), which was steam sterilized.

The roughness (arithmetical mean height, or Sa), maximum height (St) and skewness (Ssk) of the rod surface topographies was measured with a confocal microscope (µsurf explorer, NanoFocus AG, Germany) at 20× magnification, and a Gaussian filter with a cut-off wavelength of 30 µm was used. The measurements were performed on three samples, with three measurements per sample.

### 2.4. 3D Oral Mucosa Models

To produce the engineered connective tissue layer, a solution of 10× concentrated DMEM, supplemented with FBS 8.5% (*v*/*v*); L-glutamine 2 mM; and reconstitution buffer, consisting of sodium bicarbonate (22 mg/mL) and 4-(2-hydroxyethyl)-1-piperazineethanesulfonic acid (20 mM), was mixed with 5 mg/mL rat-tail type I collagen (R & D system, United Kingdom) and neutralized by 1 M sodium hydroxide to pH 7.4, while keeping everything on ice. Then 1 mL of the mixed cell suspension was added to the neutralized collagen solution at concentrations of 2 × 10^5^ fibroblasts and 1 × 10^5^ THP-1 monocytes per model (to simulate inflammatory status in the connective tissue layer), and 1 mL of the cell-populated collagen solution was transferred into cell culture inserts (0.4 µm pore size, Millipore) and incubated at 37 °C for 2 h until gel formation. The substrates were then submerged fully in complete DMEM for 3 days. Finally, 1 × 10^6^ keratinocytes were added onto each model and maintained in a submerged culture for 3 days in Green’s medium, and then were slightly raised to the air–liquid interface and further cultured for 3 days to induce some epithelial differentiation before insertion of the implants.

### 2.5. Implant/Abutment Insertion

**Figure 1:**
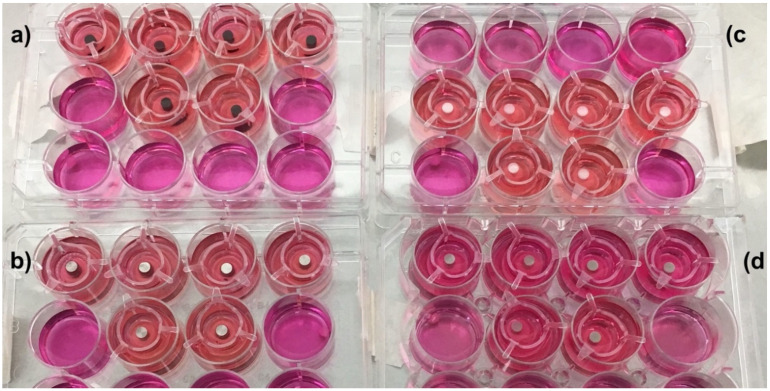
The engineered mucosa models with inserted (**a**) titanium–zirconium alloy with sandblasting and acid etching (TiZr-SLA), (**b**) machined (M) TiZr (TiZr-M), (**c**) zirconia (ZrO_2_)-M, and (**d**) polyether ether ketone (PEEK)-M rods.

TiZr-SLA, TiZr-M, ZrO_2_-M, and PEEK-M rods were inserted into the center of tissue engineered oral mucosa following a 4 mm punch biopsy. The tissue models were further cultured in the presence of the implants and abutments (Figure 1) for 72 h in Green’s medium.

### 2.6. Stimulation of the 3D Oral Mucosa Models to Induce Inflammation

Lipopolysaccharides (LPSs) of *Escherichia coli* (*E. coli*, 10 μg/mL) and tumor necrosis factor (TNF)-alpha (25 ng/mL) (Sigma-Aldrich, Dorset, United Kingdom) were added to the culture medium to simulate local inflammation conditions in the 3D oral mucosal models. The 3D oral mucosal models were incubated in a serum-free culture medium at 37 °C and 5% CO_2_.

### 2.7. Multiple Endpoint Analyses

After completion of the culture period, the tissue models were processed for multiple-endpoint analysis, as described below:

#### 2.7.1. Tissue Viability Pull Test

The soft tissue attachment of the oral mucosa to the implant surface was evaluated using a mechanical–biological approach, as described in our previous study [10]. The inserted implant discs were pulled off from the engineered mucosa after 72 h in culture. Subsequently, the amount of attached epithelial cells on the implants was assessed using the PrestoBlue tissue viability assay, which uses a non-toxic reagent and allows continuous monitoring of tissue viability. The PrestoBlue dye (Biosource, Camarillo, CA, United States) was added to the implant rods, diluted at a ratio of 9:1 (*v*/*v*) in the culture medium. The plates were then incubated for 3 h at 37 °C and 5% CO_2_. After the incubation period, triplicate samples (200 µl) were taken and placed into 96-well plate, and the fluorescence intensity was measured using a fluorescent plate reader (Infinite 200 PRO, Tecan Trading AG, Switzerland) at an excitation wavelength of 530 nm and an emission wavelength of 590 nm.

#### 2.7.2. Histological Processing

The 3D oral mucosal models were fixed in 10% (*v*/*v*) formalin solution for 24 h, and the specimens were processed for cryosectioning following the removal of the implant rods. Then 15 µm sections were prepared and stained with haematoxylin and eosin (H&E), according to a standard protocol. Histological slides were examined using a light microscope equipped with a digital camera.

#### 2.7.3. Scanning Electron Microscopy

Scanning electron microscopy (SEM) was used to visualize the epithelial cells’ attachment onto the implant surface. The implants were fixed with 10% formalin and 2% osmium tetroxide, following a graded series of ethanol dehydration and overnight air drying. When dry, the specimens were mounted on an aluminum stub, attached with a carbon sticky tab and Let-C mountant, and coated with approximately 25 nm of gold in an Edwards S150B sputter coater. The samples were examined using Tescan Vega3 SEM at an accelerating voltage of 15 kV (Electron Microscopy Facility, BioMedical Sciences EM Unit, University of Sheffield).

#### 2.7.4. Statistical Analysis

Mean values and standard deviations were calculated for quantitative continuous data. Statistical analysis was carried out with one-way ANOVA, followed by Tukey’s test using Minitab Statistical Software (Minitab Inc. Pennsylvania, United States), for which *p*-values less than 0.05 were considered as statistically significant.

## 3. Results

**Figure 2:**
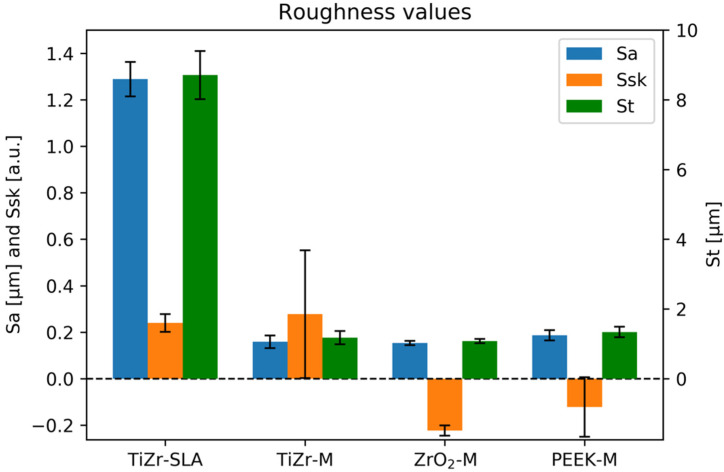
The arithmetical mean height (Sa), maximum height (St), and skewness (Ssk) of the surface topographies of TiZr-SLA, TiZr-M, ZrO_2_-M, and PEEK-M rods.

Figure 2 presents the results of the rod surface roughness measurement (*n* = 3, three rods, and each rod was measured at three random points). The results show that the *Sa* value was the highest for the TiZr-SLA rods compared to the TiZr-M, ZrO_2_-M, and PEEK-M rods.

**Figure 3:**
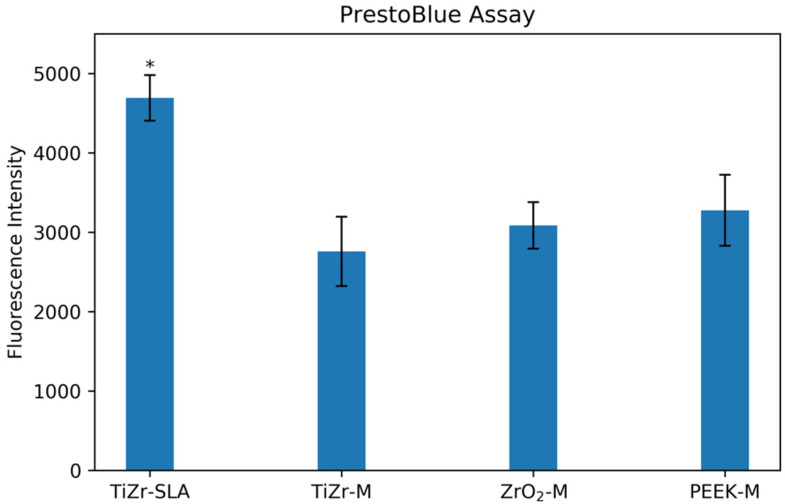
Cell viability, expressed as fluorescence intensity (590 nm), for the TiZr-SLA, TiZr-M, ZrO_2_-M, and PEEK-M rods. The error bars represent SE. * The TiZr-SLA group was significantly higher than the TiZr-M, ZrO_2_-M, and PEEK-M groups (*p* < 0.05).

Figure 3 presents the results of the pull-test for soft tissue attachment of the oral mucosa to different implant surfaces, measured by the PrestoBlue viability assay. The results demonstrate an almost two-fold, statistically significant increase of cell viability on the TiZr-SLA rods compared to the TiZr-M, ZrO_2_-M, and PEEK-M rods (*p* < 0.05).

**Figure 4:**
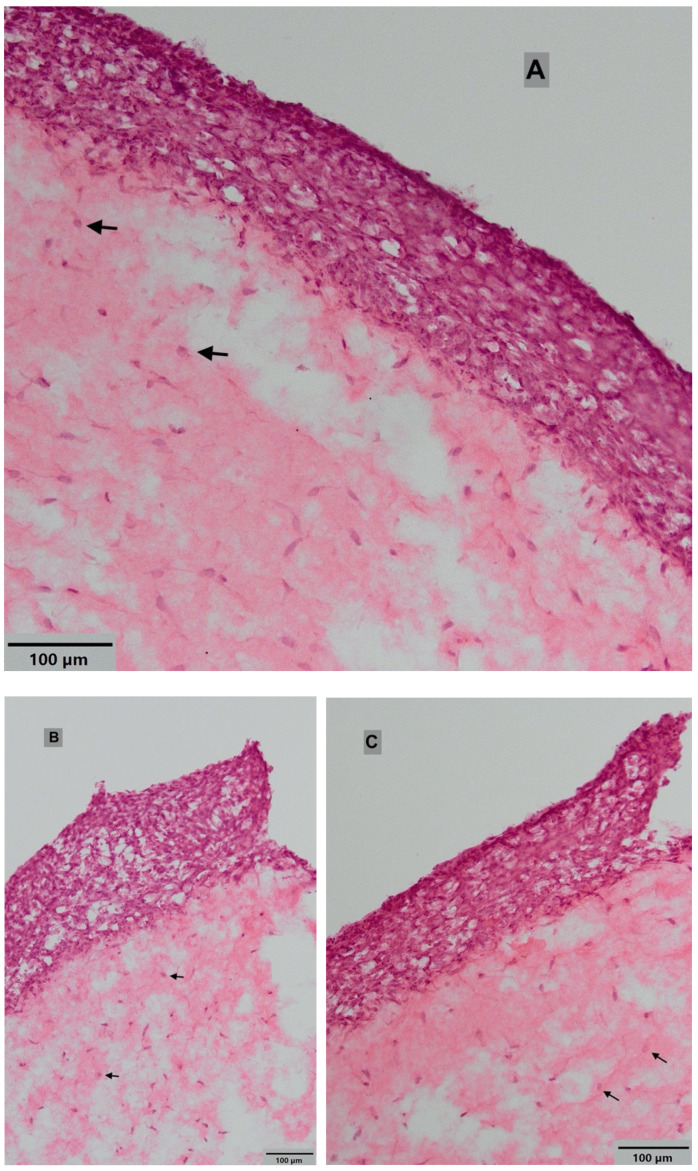
Histological views of oral mucosal models at (**A**) the center and (**B**) and (**C**) the edge of the oral mucosa at the biopsy interface. Black arrows show the monocytes in the connective tissue layer. Images were taken using light microscope at 10× magnification (haematoxylin and eosin (H&E) staining).

Figure 4 represents microscopic views of H&E-stained histological sections of the oral mucosa models. Figure 4A shows the center of the oral mucosa model, and Figure 4B,C show the edge of the mucosa at the biopsy interface. The images indicate the presence of a multi-layered, stratified oral epithelium, as well as an underlying connective tissue with a presence of fibroblasts and monocytes (black arrows).

**Figure 5:**
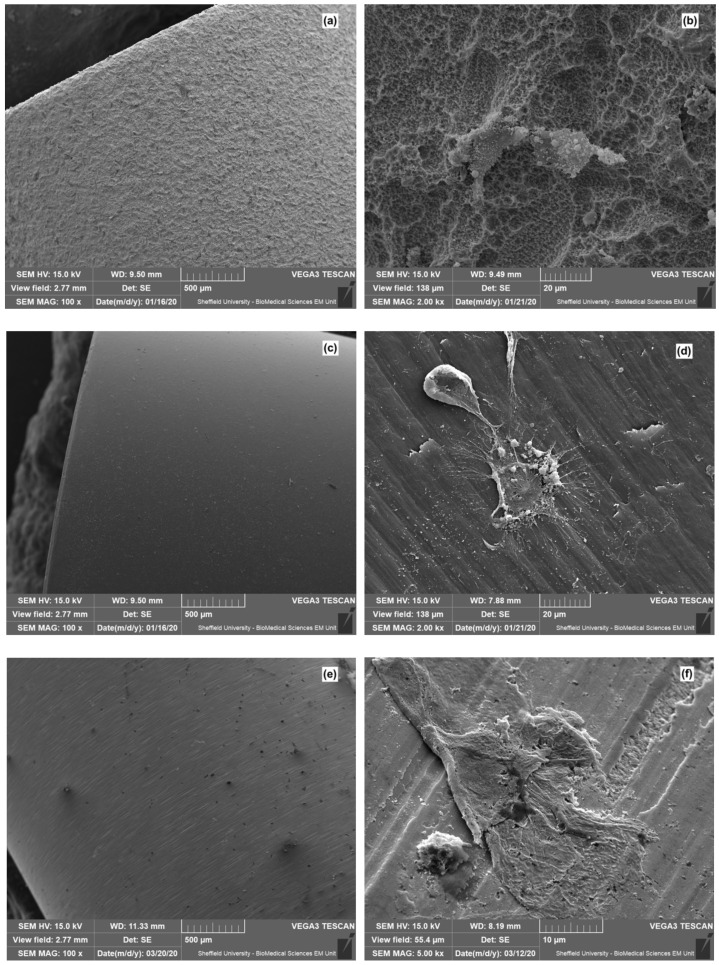

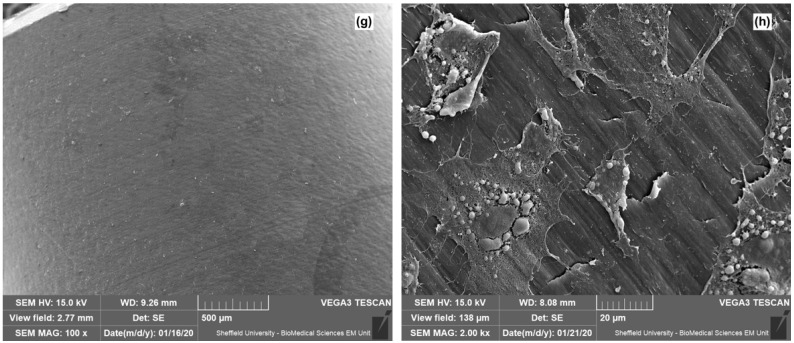
The scanning electron microscopy (SEM) micrographs of the (**a**) TiZr-SLA surface, (**b**) TiZr-SLA surface with cell attachment, (**c**) TiZr-M surface, (**d**) TiZr-M surface with cell attachment, (**e**) ZrO_2_-M surface, (**f**) ZrO_2_-M surface with cell attachment, (**g**) machined PEEK surface, and (**h**) machined PEEK surface with cell attachment.

Figure 5 presents the SEM micrographs of the oral mucosal cells’ attachment onto different implant surfaces. The micrographs show clear differences between the implant surfaces. The most distinct is the TiZr-SLA surface (Figure 5a,b), with its characteristic honeycomb structure. The other implants—TiZr-M, ZrO_2_-M, and PEEK-M—had very similar surface characteristics: smooth surfaces with elongated grooves that were acquired during manufacturing process. All implants showed the potential for cell attachment (Figure 5b,d,f,h). However, the cells’ morphology appeared to be different on each surface. They were flat on the TiZr-M, ZrO_2_-M, and machined PEEK surfaces (Figure 5d,f,h), and more three-dimensional on the TiZr-SLA surface (Figure 5b).

## 4. Discussion

There has been an increased interest in studying the biological responses of tissue-engineered human oral mucosa to different dental implant surfaces in recent years.

Ingendoh-Tsakmakidis et al. [22] investigated the response of peri-implant oral mucosa to oral bacterial biofilms using an in vitro, peri-implant, mucosa–biofilm model. In this study, a titanium disc (implant material) was inserted into the engineered connective tissue layer, consisting of collagen-embedded human gingival fibroblasts (HGFs), and then oral keratinocytes (OKF6/TERT-2) were added around the titanium on top of the fibroblast–collagen gel. This procedure is slightly different than the clinical situation, where the implant is inserted following a full-thickness mucosal biopsy or incision through both epithelium and the connective tissue layers. The authors investigated the transcriptional activity of heat shock protein (HSP70) genes and the inflammatory response reflected by cytokine levels of interleukin (IL)-6, IL-8 (CXCL8), and monocyte chemoattractant protein-1 (CCL2). This model, however, lacked any inflammatory cells, which are a key element of the oral mucosal immune response.

Shang et al. studied the interaction between multi-species oral commensal biofilm and 3D reconstructed human gingiva (RHG). They showed an increase in the secretion of inflammatory and antimicrobial cytokines, including IL-6, CXCL8, CXCL1, and CCL20, which had a positive influence on the host tissue by promoting extensive stratification and enhancing antimicrobial defense [23].

Roffel et al. have developed an in vitro implantation model of oral mucosa for assessment of molecular interactions with implant abutment surfaces [24]. Although immunohistochemical analysis techniques were used to characterize soft tissue attachment to the abutment surfaces, their model lacked normal oral cavity environmental conditions, such as the presence of bacterial products that can affect soft-tissue attachment to the implants and abutments. Furthermore, the 3D model was based on fibroblasts and keratinocyte cell lines only, without the presence of any immune cells, which can affect the cell attachment to the abutment surface during the inflammatory healing phase. In our pilot study, bacterial LPSs and TNF-alpha were added to the culture media to further simulate the clinical situation; however, any environmental deoxyribonucleic acid (eDNA) or other bacterial components were not included in the present study, and can be considered in future studies of the assessment of gingival soft tissue to different metal, ceramic, and plastic implant and abutment surfaces.

Titanium (Ti) is the most commonly used material for the fabrication of dental implants, which are used for the oral rehabilitation of patients with missing teeth [25,26,27]. This is due to its resistance to corrosion, mechanical properties, and high biocompatibility, especially the ability for osseointegration with surrounding bone tissue [28,29,30]. However, reduced diameter of implant has been associated with an increased risk of fatigue fracture [31,32,33]. Zirconium-containing titanium alloys have the advantage of adequate mechanical strength and enhanced corrosion resistance in biological fluids [34,35,36,37]. Several clinical studies [28,38,39] have investigated the commercially available TiZr implants (Roxolid, Institut Straumann AG) with similar SLA-active surface treatments. Chiapasco et al., evaluated the success rates of 51 small-diameter TiZr implants placed in horizontally deficient ridges in 18 patients [38]. The results indicated a high success rate for TiZr implants, ranging from 95.2% to 100%. The main limitation of titanium and its alloys is suboptimal aesthetic properties, especially when there is thin gingival biotype in the aesthetic zone. In these circumstances, zirconium and PEEK abutments would be more aesthetic alternatives, which can be considered by the clinicians to achieve ideal aesthetic clinical outcome.

PEEK is a thermoplastic and semi-crystalline polymer with a high melting temperature. The elastic modulus (EM) of PEEK is 3.6 GPa, and the addition of carbon fibers can increase it to 18 GPa, close to the EM of cortical bone (15 GPa) [40,41,42]. PEEK is highly biocompatible, has a non-metallic taste, and does not cause attrition of the opposing natural teeth. It can be polished easily, and has low plaque retention and high wear resistance. It has been used as a framework material for removable partial dentures, as an alternative to cobalt chromium alloys. Using PEEK material often results in production of lightweight and hygienic prostheses with high patient satisfaction [43].

This pilot study demonstrated the feasibility of qualitative and quantitative assessment of oral soft tissue attachment to different metal, ceramic, and polymer implant surfaces, using an inflamed, 3D, tissue-engineered, human oral mucosal model.

Our initial findings indicate a clear difference in cell attachment depending on the type of implant surface topography. The rods were removed during the pull test by holding them with a pair of tweezers. The cells that were still attached to the implants at the interface were quantified using the PrestoBlue viability assay (Figure 3). The results showed an increase in the cell viability on the TiZr-SLA surface compared to the other groups, indicating a higher number of cells remaining attached and/or higher metabolic activity, which could contribute to a favorable cell growth environment.

The histological data shows that the inflamed oral mucosa model developed in this study closely mimicked the in vivo situation. The 3D dimensional structure consisted of the connective tissue collagen layer, containing fibroblasts and monocytes, and the distinct epithelial layer, with multi-layered stratified oral keratinocytes. However, it remains to be established which cells have a greater tendency for attachment to the implants. In our future experiments, we are interested in identifying the type of cells (fibroblasts or keratinocytes) that remain attached on different implant surfaces after the pull test, in order to further explore the nature of the soft tissue attachment on various implant and abutment surfaces.

The SEM findings provide supporting evidence to the PrestoBlue viability results that mucosa models formed an attachment onto all implant surfaces (Figure 5) tested in this study. As some cells remained attached to the implants after removing the implants from the oral mucosa models, it can be interpreted that the bond between the cells and implant surfaces were stronger than the bond between the cells within the oral mucosal model. In line with our findings, Jung et al. [44] recently evaluated the adhesion of different cell lines, including gingival fibroblasts and osteoblasts, to different titanium and zirconia implant surfaces. They showed that the surface texture of the implants significantly affected the cell morphology and cytoskeleton. Rough surfaces resulted in higher expression of Actin beta and vimentin by the cells. Surface structure significantly affected the bond strength between the cell and the implant material. However, the cell type and implant material regulated the type of cell and the implant binding. On the other hand, Durkhan et al. [45] investigated the vitality of oral keratinocytes and gingival fibroblasts, the number of adhered cells, and their bond strength to nano-structured titanium implant surfaces. They concluded that the level of cell adherence and vitality of the soft tissue were similar for commercially pure titanium and two anodically oxidized implant surfaces. However, the addition of streptococci bacteria significantly reduced the number of oral keratinocytes on all types of the implant surfaces.

The SEM images provide a possible explanation for the viability assay results where the TiZr-SLA implant group presented higher fluorescence readings. The surfaces of TiZr-SLA implants are clearly different than TiZr-M, ZrO_2_-M, and machined PEEK. They resemble a honeycomb structure, with a higher surface area due to roughness and the presence of crevices and surface irregularities, compared to the smooth surfaces with elongated grooves of machined implants. The measurement of roughness of the rods (Figure 2) confirms those findings, as the Sa value was the highest for the TiZr-SLA implants. These irregularities could provide more effective cellular attachment and/or more favorable environment for cellular metabolism. This could be manifested by the different cell morphology, which was three-dimensional on the TiZr-SLA surfaces compared to the flat cellular structures on the TiZr-M, ZrO_2_-Mm and machined PEEK surfaces.

Giannasi et al. [46] investigated the proliferation, adhesion, and spreading of monolayer human primary gingival keratinocyte cells and epithelial progenitor cells on machined, Ti-Unite, and SLA implant surfaces, using a combination of imaging and biochemical cell viability testing. The authors demonstrated that cell adhesion was significantly higher on machined and Ti-Unite surfaces than on the SLA implant surfaces. In another study using monolayer primary gingival fibroblasts and keratinocytes, Xu et al. [47] reported that applying anodic oxidation on a micro-roughened, selective laser melted titanium surfaces, and subsequent calcium phosphate nanoparticle embedding resulted in enhanced bioactivity when compared to mechanical polishing. Perez-Diaz et al. [48] demonstrated that the surface morphology of titanium can influence fibroblast cell adhesion and viability differentially and independently of the roughness parameters. A sintered titanium powder surface had the highest bioactivity compared to machined, SLA, and laser-treated surfaces. It has also been shown that aging samples in an autoclave can enhance cell proliferation and adhesion and reduce biofilm adhesion on titanium surfaces more than that of zirconia [49]. It is important to note that the results from the in vitro studies described above were based on monolayer cell culture systems, and can be different when 3D, multi-layered, full-thickness oral mucosal models are used. In previous studies by Chai et al., the interactions between implants and oral soft tissues have been investigated at the interface, using 3D, tissue-engineered human oral mucosa models [10,11,12,13]. Microscopic imaging has revealed that cells attached to titanium surfaces in both a “pocket” and “non-pocket” manner, depending on the epithelium’s contact angle with the implant surface. It was also shown by TEM examination of ultrathin (electropolished) sections that the cells grew into the irregular and porous surfaces. Furthermore, the evidence of hemidesmosome-like structure formation on titanium surface topographies have been reported [14].

In a combined in vivo and in vitro study, Atsuta et al. [50] evaluated the effect of surface roughness on peri-implant epithelial downgrowth and seal. Although there was no difference between the test groups at 4 weeks, after 16 weeks of implantation in rats, rough-surfaced implants formed a weak epithelial seal and showed significantly less peri-implant epithelial down-growth compared to the machine-surfaced implants. The ability of the cells to grow into the irregularity of titanium surfaces has been demonstrated in two other in vivo studies [51,52]. Deporter et al., presented histological data from a dog study, using a porous-surfaced titanium implant system with a transgingival collar to encourage the ingrowth and attachment of gingival soft tissue. Although connective tissue attachment occurred for some implants in the porous collar region, bacterial contamination deteriorated the prognosis of most of the implants [51]. Schroeder et al. reported the results of animal experiments on monkeys, demonstrating the functionally-orientated insertion of connective tissue fibers into the titanium-sprayed surface and signs of epithelial cell adhesion when implants are placed in keratinized gingiva [52]. Earlier animal studies have shown that hemidesmosomes can form as early as 2 days after surgery [53].

In our current study, the pull tests were performed 3 days after rod implantation to enable early analysis of differential soft tissue attachment to the implant surfaces, which indicated that soft-tissue attachment can be initiated within several days following implantation. It has been reported that epithelial healing after periodontal treatment can be completed within 7–14 days. However, establishment of the biological width and maturation of the barrier function around dental implants may take 6 to 8 weeks [54].

Further experiments on the release of different pro-inflammatory cytokines (such as interleukins 1, 4, 8, and 10 from the oral mucosal models), immunohistochemical characterization of the interface (including cytokeratins 4, 16, and 19, collagen IV, and laminin-5), and investigation and comparison of different commercially available implants and abutment surfaces are currently underway to further improve our knowledge and understanding of the mechanisms of inflamed oral soft tissue attachment onto different implant and abutment surfaces.

## 5. Conclusions

A full-thickness, 3D, tissue-engineered human oral mucosal model can be assembled successfully using oral fibroblasts, OKF6/TERT-2 keratinocytes, and THP-1 monocytes. The 3D oral mucosal model combined with LPSs and TNF-alpha-induced inflammation, and has the potential to be used as a suitable in vitro test system for the visualization of implant soft-tissue attachment using histology and SEM, as well as its quantification with the PrestoBlue tissue viability pull test. The results of our pilot study indicated greater soft tissue attachment to TiZr-SLA implants compared to machined TiZr, ZrO_2_, and PEEK surfaces. Further experiments are underway to investigate the differential adhesion and proliferation of different layers of the gingival mucosa to different implant and abutment surfaces.
